# Animal Research: Charting the Course for FAS

**Published:** 1994

**Authors:** Howard C. Becker, Carrie L. Randall, Allen L. Salo, Jocelynn L. Saulnier, R.T. Weathersby

**Affiliations:** Howard C. Becker, Ph.D., is an associate research career scientist at the Ralph H. Johnson Veterans Affairs Medical Center and an associate professor in the Department of Physiology and the Department of Psychiatry and Behavioral Sciences, Medical University of South Carolina, Charleston, South Carolina. Carrie L. Randall, Ph.D., is a research career scientist at the Ralph H. Johnson Veterans Affairs Medical Center and a professor in the Department of Physiology and the Department of Psychiatry and Behavioral Sciences, Medical University of South Carolina, Charleston, South Carolina. Allen L. Salo, Ph.D., is a postdoctoral fellow in the Department of Psychiatry and Behavioral Sciences; Jocelynn L. Saulnier, B.Sc., is a graduate student in the Department of Physiology; and R.T. Weathersby, Ph.D., is a postdoctoral fellow in the Department of Psychiatry and Behavioral Sciences, Medical University of South Carolina, Charleston, South Carolina

## Abstract

Animal models of FAS have allowed researchers to study the mechanisms behind alcohol’s deleterious effects on fetal development. Such models have helped verify hypotheses based on studies of children with FAS and uncover new features of FAS not evident in humans.

Since the “formal” identification of fetal alcohol syndrome (FAS) in 1973, interest in developing and studying animal models of FAS has swelled, giving rise to a thriving area of research. The development of animal models has provided an invaluable research tool for characterizing and advancing the study of alcohol’s adverse effects on the developing fetus. By employing several species (including nonhuman primates) and a variety of methodologies and experimental approaches, animal research has played a major role in advancing knowledge of the many immediate and long-term deleterious consequences that follow prenatal alcohol exposure.

Animal research, performed under controlled laboratory conditions, substantiates hypotheses formed by observing humans with FAS; in other cases, it helps form new hypotheses. Using animal models, researchers have discovered detailed brain abnormalities related to FAS, identified critical periods and critical doses of alcohol exposure, and identified alcohol-induced biochemical changes in the pregnant animal or her fetuses. These issues are beyond the bounds of clinical FAS research with humans either for ethical reasons or because researchers are unable to exercise the type of control over the genetic background and environment that is needed for a truly accurate experiment.

After discussing in more detail the benefits of FAS animal models and some related general findings, this article will highlight some of the specific observations made by basic animal research in the last 20 years.

## Animal Research

### Benefits of Animal Models

Animal studies allow researchers a degree of control impossible with research on humans. Researchers can control the genetic background of the parent animals as well as certain environmental factors. Rigorous control may be exerted over variables such as amount of alcohol (i.e., alcohol “dose”), pattern of alcohol consumption, duration and timing of alcohol exposure during pregnancy, and maternal nutritional status. For example, to identify a threshold dose of alcohol above which fetal damage occurs, alcohol can be administered on particular days or weeks of pregnancy, or the amount of alcohol given can be varied from animal to animal. Further, using animals with different genetic backgrounds may provide insight into the possible contribution of genetic factors that may govern susceptibility to alcohol-related damage.

One of the first issues tackled by animal researchers was whether alcohol, itself, was responsible for the constellation of birth defects labeled FAS. Alcoholic women frequently abuse tobacco, have poor health, and are malnourished. Any of these factors could result in birth defects, and skeptics questioned the focus on alcohol ingestion alone in the coining of the term “fetal alcohol syndrome.” Thus, animal models were used to determine if alcohol alone was a teratogen—a substance capable of causing birth defects.

Almost simultaneously, two different laboratories reported that alcohol administration to pregnant mice resulted in smaller offspring and birth defects similar to those reported in humans with FAS (Chernoff 1977; Randall et al. 1977). These studies controlled for polydrug use, maternal health, and nutritional factors, indicating that alcohol, per se, is teratogenic to humans and mice.

Since the publication of the studies in mice, the teratogenic effects of alcohol have been demonstrated in many other species, including nonhuman primates (National Institute on Alcohol Abuse and Alcoholism [NIAAA] 1990, 1993). However, the majority of the research generated with animals has used either mice or rats. Mice have been used primarily to examine alcohol-induced birth defects (structural malformations), whereas rats have been used primarily for studying alcohol-induced behavioral deficits. Indeed, the structural and functional defects observed in these rodent models of FAS are remarkably similar to those identified in human clinical studies (Driscoll et al. 1990).

Determining alcohol’s threshold dose and mechanism of action is more complicated than demonstrating that alcohol is a teratogen. It is highly unlikely that researchers will identify a single threshold dose or safe limit of prenatal alcohol exposure; a unique, circumscribed, and sensitive period of development; or one mechanism of action for the various effects of prenatal alcohol exposure in humans.

To make the task even more complicated, it is likely that different aspects of development may be sensitive to different levels of alcohol. Despite the complexity of the issues, scientists have made inroads into understanding this formidable disorder. Since the identification of FAS two decades ago, animal research has produced a wealth of general information regarding the effects of prenatal alcohol exposure on the fetus. Some of these findings are summarized below.

### General Findings

#### Critical Periods

By varying when during pregnancy females are given alcohol, animal research has demonstrated that the specific type of birth defect produced depends on the system(s) in the fetus undergoing development at the time of alcohol exposure (Becker 1992; Randall 1987; Webster 1989). Organ systems are most vulnerable to damage by alcohol during the period of most dynamic development. For example, heavy alcohol exposure during the period of craniofacial development (which roughly corresponds to the third and fourth weeks of human pregnancy) will affect the structure of the facial features but not the structure of the kidney, an organ system that develops around the sixth week of human pregnancy (Webster 1989). This type of exposure and damage can be considered a “binge”-type model.

As would be expected, heavy alcohol consumption throughout pregnancy, as opposed to occasional binges, risks the entire constellation of structural defects involving several major organ systems and including growth retardation and brain abnormalities (NIAAA 1990, 1993). This pattern is more consistent with what is known as FAS.

The complexity of alcohol-related damage is perhaps best illustrated when considering the brain and its sensitivity to alcohol during development. Unlike other organs, the brain is one of the first organ systems to begin to develop and the last to be completed. Thus, the brain appears to be sensitive to the adverse effects of alcohol throughout its development and over all trimesters of pregnancy. Animal research has revealed that some brain regions are more sensitive to the teratogenic actions of alcohol and, further, that some cell populations are more vulnerable to alcohol insult than others even within a particular brain region (West 1986).

Thus, critical periods of vulnerability to the harmful effects of alcohol on brain development appear to vary among various regions of the brain. This pattern of damage may explain the wide array and complex pattern of behavioral and neurological abnormalities that have been observed in human and laboratory animal offspring exposed to alcohol at different times during fetal development.

#### Critical Doses

Animal models have shown that peak blood alcohol level rather than total amount of alcohol consumed may represent the “critical dose,” or threshold, of alcohol above which an adverse effect will be seen (West et al. 1990). This finding implies that drinking *pattern* is critical. Rapid consumption of alcohol in a short period of time will result in a higher blood alcohol level than sipping the same amount of alcohol slowly over a long period of time. These results underscore the importance, when studying humans, of researchers asking women about their pattern of drinking in addition to the total number of drinks, as the former may have a greater negative impact on pregnancy outcome.

### Comparing Symptoms of FAS in Animal Models and Humans

**Figure 1:**
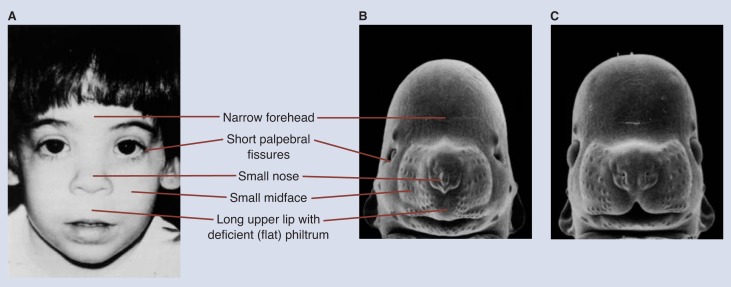
Similarities of facial defects found in (A) humans and (B) mice exposed prenatally to alcohol. Panel C shows a control mouse fetus not exposed to alcohol. Photograph courtesy of Kathy K. Sulik.

The symptoms found in animal models are remarkably similar to those observed in humans (Driscoll et al. 1990). This similarity applies to physical defects (as illustrated in figure 1) and behavioral effects (table 1). In addition, the deleterious effects of in utero alcohol exposure in animal models, as in humans, have been shown to exist along a continuum. Both the full-blown syndrome (FAS) and the partial expression of the syndrome, which has been termed “fetal alcohol effects” (FAE), have been seen in alcohol-exposed animals. With adequate animal models, researchers can begin to identify problems not yet identified in humans and the mechanisms of alcohol’s effect.

### Birth Weight and Growth Patterns

Retarded growth in utero and after birth is a hallmark of FAS and has been reported in a wide variety of species prenatally exposed to alcohol (NIAAA 1990, 1993). In many cases, body weight and body length deficits and small heads (microcephaly) were dose related and observed after various exposure patterns, although exposure to alcohol during the equivalent of the second and third trimesters appeared to be most critical for this pregnancy outcome (Becker 1992; Randall 1987; West 1986). In some instances, there was evidence of “catchup” in growth as the animals matured; in others, the growth retardation extended into adulthood (Becker 1992). Some recent reports in mice have indicated that the deficits in growth may not be apparent until adolescence and early adulthood (Becker 1992).

Animal models allow the use of cross-fostering techniques to minimize the possibility that deficient growth patterns are caused merely by postnatal factors, such as poor maternal behavior, rather than in utero alcohol exposure, per se. With cross-fostering, alcohol-exposed offspring are raised by control mothers, and control offspring are raised by alcohol-exposed mothers. Data from animal studies indicate that both reduced birth weight and postnatal growth retardation can be attributed primarily to prenatal alcohol exposure.

### Major Organ Systems

As in humans, animal models have demonstrated a wide variety of organ anomalies after acute and chronic exposure to alcohol (Becker 1992; Randall 1987). The organs are most sensitive to damage by alcohol exposure during organogenesis, a period when they are first developing. In rodents, the period of organogenesis includes the second week of a 20- to 21-day gestation period (Becker 1992; Webster 1989). This roughly corresponds to weeks 3 to 8 in human pregnancy.

As mentioned earlier, the time of alcohol exposure within this period determines which developing organ system(s) will be affected. Animal studies have modeled these possibilities through careful control of the timing of alcohol exposure during embryonic/fetal development (Becker 1992; Webster 1989).

#### Craniofacial Defects

The presence of characteristic defects of the face and head is important for the diagnosis of FAS in humans. Animals exposed to alcohol during early organogenesis are born with a pattern of craniofacial defects that are remarkably similar to the facial features of children with FAS (figure 1) (Sulik et al. 1981; NIAAA 1990, 1993). Further examination of these animal fetuses revealed other brain malformations, suggesting that the craniofacial defects may indicate brain abnormalities (Kotch and Sulik 1992). Indeed, a positive relationship has been noted between the severity of craniofacial defects and mental disability in children with FAS (Streissguth et al. 1991; NIAAA 1990, 1993).

#### Skeletal Anomalies

Exposure to alcohol during organogenesis results in skeletal malformations in laboratory mice, especially limb defects (Becker 1992). Limb malformations primarily have involved the forelimbs, with incomplete growth of one or both forelimbs and various types of defects of the digits on the limbs. The digit defects have been attributed to cell death of the limb buds following alcohol exposure. Other skeletal anomalies reported include those of the vertebrae, sternum, and ribs (Becker 1992). The pattern of skeletal defects observed in mice is similar to that observed clinically in humans.

#### Cardiovascular Anomalies

Animals exposed to alcohol at a time that roughly corresponds to as early as weeks 3 to 4 of human pregnancy have had cardiovascular malformations that are similar to those reported in children with FAS (Becker 1992; Webster 1989). Most notably, they include malformations of the heart itself and of the vessels leading to and from the heart.

#### Urogenital Anomalies

In both experimental animals and humans, the developing kidney has been shown to be sensitive to alcohol-induced damage (NIAAA 1990, 1993). Kidney defects were observed in children with FAS only after they were first identified in animals. Renal malformations typically have included structural defects of the kidney and the ureter, which carries urine from the kidney to the bladder. Abnormal sexual development and function also have been documented in animals prenatally exposed to alcohol. Many of these effects may be attributed to alcohol-induced alteration of sex hormones both at the level of the brain and in the sex organs (discussed below).

### Sensory and Motor Effects

A variety of sensory and motor deficits have been identified in children with FAS and modeled in studies with experimental animals. These include visual, auditory, balance, and motor coordination problems.

#### Visual Deficits

Defects involving the eyes and the visual process are common in children with FAS and typically result in impaired visual acuity and nearsightedness. The most common defects are small eyes, drooping eyelids (ptosis), short eye openings, crossed eyes, a reduction in the number of optic nerve axons, and abnormal vasculature in the retina. A similar pattern of eye anomalies has been found in animal models (NIAAA 1990, 1993). Investigations of the underlying cause of these defects have shown that embryonic alcohol exposure results in an increased rate of cell death and impaired cell replication in the retina as well as reduced myelination (insulation) of the nerve that connects the eye to the brain. The developing eye appears to be sensitive to alcohol-induced damage.

#### Auditory Deficits

Children with FAS show a high prevalence of hearing impairment related to structural damage of the ear during its development. Research with animals has demonstrated that prenatal alcohol exposure can have deleterious effects at various levels of the auditory system. For example, rats prenatally exposed to alcohol have been found to have hearing loss resulting from both abnormal electrical activity in the brainstem and disruptions in auditory processing at the level of the cortex (NIAAA 1990, 1993).

#### Balance and Motor Deficits

Children with FAS have a wide range of balance and motor coordination deficiencies, some of which persist well into childhood (documented through 12 years of age). They include problems with balance and gait, muscle tremors, and deficits in both gross and fine motor function. Rats prenatally exposed to alcohol have shown delayed development of motor reflexes, deficits in performance of tasks that require motor coordination, and alterations in walking pattern (NIAAA 1990, 1993). Many of these motor problems suggest damage to the cerebellum, a part of the brain that plays an important role in controlling motor coordination. Rat studies have confirmed this speculation, as have autopsies of humans with FAS (West 1986).

## Effects on Behavior

### Neonatal and Regulatory Effects

#### Fetal Movement

The deleterious effects of alcohol exposure have been well characterized in offspring after birth, but relatively little is known about the behavior of the alcohol-exposed fetus in utero. Recent procedures involving direct observation of rat fetal activity in utero showed a substantial reduction in overall activity and movements compared with non-alcohol-exposed fetuses (NIAAA 1990, 1993). Such alcohol-induced suppression of prenatal activity also has been shown regarding breathing movements and brain activity in near-term fetal sheep and in the fetuses of pregnant women who consumed alcohol (NIAAA 1990, 1993).

Altered fetal activity and reduced fetal movement have been associated with profound effects on morphological development in humans and animals. Therefore, alcohol-induced suppression of movement in the developing fetus may be a contributing factor in FAS.

#### Feeding Behavior

Human newborns prenatally exposed to alcohol are not good feeders. They are easily distracted and fatigued when suckling, and, even when they do feed, they have a weak, irregular suckling response. Similar feeding abnormalities have been found and systematically studied in rodents prenatally exposed to alcohol (Driscoll et al. 1990). Rat studies suggest that newborn rats that do not nurse successfully may have impaired ability to detect smell cues, thereby hindering the pups’ ability to locate the mother’s nipples; difficulty in coordinating motor behaviors; or a dampened level of arousal to stimuli related to attaching to the nipple.

These findings suggest that altered feeding behavior may be related to the growth deficits observed postnatally. If so, basic research efforts can identify ways to improve feeding behavior that may have clinical application.

### Long-Term Behavioral Effects

FAS was diagnosed initially in young children, and although abnormal behaviors were observed at birth and during the neonatal period, it was not possible to predict if behavioral problems would get better as the children got older, if they would stay the same, if different types of abnormal behaviors would be observed with maturation, or if the children could be taught to utilize strategies that compensate for their behavior. Given that animals mature significantly faster than humans do, the long-term effects of prenatal exposure to alcohol could be more easily examined in animal models.

Now that study populations of humans with FAS are growing older, researchers are finding that abnormal behaviors do persist. The animal models can be used to evaluate the effectiveness of interventions, particularly drug therapies, that try to alleviate abnormal behaviors.

In animals, many of the behavioral effects of prenatal alcohol exposure have been shown to diminish with age (NIAAA 1990, 1993). It has been proposed that rather than irreparably altering behavior, alcohol merely retards brain development, with eventual catchup or compensatory behaviors producing more normal development. Recent data, however, have shown the reemergence of some deficits in older animals, suggesting either that the compensatory systems break down with age or that more complex or difficult tasks unmask persistent defects (West et al. 1990; NIAAA 1990, 1993). In particular, these deficits include impaired performance in learning and memory tasks.

These findings complement those of clinical studies that have revealed persistent attentional, cognitive, and neurobehavioral deficits in children of alcohol-abusing mothers, primarily when the testing conditions were challenging to the children (Streissguth et al. 1991).

Animal models should prove useful in identifying the types of behavioral deficits that are permanent and those that are transient developmental delays. Even more important, however, is the use of animal models to develop effective interventions to improve cognitive learning and memory, whether the interventions be pharmacological or behavioral in nature. Such information is critical in identifying special education approaches for children exposed to alcohol prenatally so that they can more fully benefit from school.

### Effects on the Central Nervous System

Behavioral problems observed in offspring exposed prenatally to alcohol, such as hyperactivity and perseveration (repetition of a mental activity with an inability to switch to another activity), poor balance and coordination, difficulty walking, and the inability to concentrate or to learn from past experiences, are all indicative of abnormal development of the brain, or central nervous system (CNS). Those CNS defects include abnormal structure and connections of brain cells and irregular communication from cell to cell.

Animal models have demonstrated relationships between abnormal behaviors and specific structural defects in the brain. For example, learning and memory deficits have been demonstrated in animals that exhibited, on post mortem examination, alcohol-induced damage in the hippocampus, a part of the brain that plays an important role in mediating these mental activities (NIAAA 1990, 1993). Also, motor coordination problems have been linked to structural defects in the cerebellum.

Such structure-function associations observed in animal models may serve to guide similar studies in human FAS/FAE populations, where more sophisticated neuropsychological tests may be combined with powerful brain-imaging techniques, such as nuclear magnetic resonance, magnetic resonance imaging, and positron emissions tomography scanning (see the article by Mattson et al. on pp. 49–52).

The discovery of altered brain chemistry in prenatally alcohol-exposed offspring also may help to explain altered behavior. Chemicals in the brain—neurochemicals—control brain function and behavior.

Neurochemical systems are involved in the expression of different types of behaviors, including feeding behavior, sleep, and even mental health problems such as depression and anxiety. Animal studies have found that several neurochemical systems are altered in animals exposed prenatally to alcohol (NIAAA 1990, 1993). The identification of specific neurochemical imbalances in animal models and of drugs that alleviate these imbalances may lead to appropriate pharmacological interventions for humans with similar behavioral problems.

### Effects on Neuroendocrine Systems

Levels of the sex hormones testosterone and estradiol are controlled by a complex feedback system that is critical to maintaining the proper balance of these hormones. The hypothalamus (the part of the brain that converts incoming electrical information from neurons into chemical, bloodborne messengers called hormones) sends hormonal messages to the pituitary (the body’s master endocrine gland). The pituitary then releases other hormones that act on specific parts of the body, such as the testes (in men) or the ovaries (in women). These target tissues release yet more hormones—testosterone and estradiol, for example—that on reaching standard levels in the blood, trigger the hypothalamus and pituitary gland to stop releasing hormonal messengers.

Disruption of any part of this loop (hypothalamus → pituitary → target tissues → hypothalamus/pituitary) can have detrimental effects. Rat models have shown that prenatal alcohol exposure alters the feedback system in both males and females by altering activity in the critical brain structures and the sex organs (NIAAA 1990, 1993). As a result, sexual maturation is delayed, sexual behavior is disrupted, hormone levels are abnormal, and reproductive function is altered.

Abnormal hormonal levels during the critical period of sexual differentiation of the brain may be expected to produce long-term effects. In the rat, this critical period corresponds to the end of gestation and extends into the first week of postnatal life (this is equivalent to the third trimester of pregnancy in humans). During this time, the production and secretion of testosterone are the primary hormonal influences that promote the masculinization of the brain in male rat offspring. Prenatal alcohol exposure blunts the normal testosterone surge in male neonates (NIAAA 1990, 1993), resulting in demasculinization, or feminization, of certain brain structures and behaviors in adult male offspring.^1^

This finding might have implications for sexual maturation in humans with FAS (Streissguth et al. 1991). It will be interesting to see if abnormal sex hormone regulation and reproductive function observed in animal models predict similar problems in humans exposed to alcohol prenatally. Given that FAS was not identified until 20 years ago, such observations should only now be seen as the newborns who were given a diagnosis of FAS in the early 1970’s reach sexual maturation and enter their reproductive years.

Another neuroendocrine anomaly observed in animal models is the biochemical response to stress in rats exposed to alcohol in utero. The reaction to stress is controlled by a feedback loop similar to that of the sex hormones, involving the hypothalamus, the pituitary gland, and the adrenal glands as the target tissue (as opposed to the testes or ovaries). Research findings have indicated a heightened response to stress in offspring exposed to alcohol prenatally (NIAAA 1990, 1993).

Animal models are being used to identify the exact location of the defect in the feedback loop. An aberrant response to stress as a function of exposure to alcohol prenatally has direct clinical implications, especially because alcohol is sometimes consumed to relieve stress. Additionally, maladaptive responses to stress would be expected to interfere negatively with daily functioning.

## Effects on the Immune System

Animals exposed prenatally to alcohol have demonstrated alcohol-induced deficiencies in the immune response that might explain the increased risk for infections observed in children with FAS. In particular, the animals showed a reduction in the number of cells that fight infection, the T-lymphocytes, which in turn increases susceptibility to infection and, possibly, cancer. Although this area of research has not been studied as extensively as some of the others discussed above, it promises to provide important clinical information related to identification and intervention so that the severity and number of infections can be reduced.

## Studies on Mechanisms of Fetal Alcohol Damage

Identifying the mechanism(s) of action by which alcohol affects fetal growth and development is one of the most important roles that animal research can play. Given the multitude of different types of defects, the involvement of all major organ systems, and the involvement of many dysfunctional neurochemical and biochemical processes, the search for the underlying mechanism(s) will not be easy. It may be that several different mechanisms are responsible for the myriad of deleterious effects that follow prenatal alcohol exposure.

Alcohol could affect fetal development in several ways: directly, by damaging and killing the developing fetal cells; indirectly, by affecting placental function in the mother; or by affecting any one of the numerous biochemical steps involved in the process of fetal development.

Animal research has found that alcohol readily crosses the placenta and can directly alter fetal development by attacking the developing cells, thereby decreasing cell size and cell number in several organ systems (NIAAA 1990, 1993). These studies do not, however, rule out other mechanisms. Indeed, research has shown that alcohol-induced damage probably results from both direct and indirect effects of the drug on the developing fetus.

Animal research in pregnant rats has shown rather consistently that alcohol can decrease the transport of essential nutritional elements called amino acids across the placenta to the fetus (NIAAA 1990, 1993; Schenker et al. 1990); similarly, glucose transport has been shown to be reduced. A reduction in transport of either of these building blocks could explain decreased fetal weight and other types of abnormal development.

It has been suggested that alcohol-induced structural defects may be a result of an alteration in a class of compounds called prostaglandins that are important for normal fetal growth and development as well as control of umbilical blood flow.^2^ An imbalance of prostaglandin levels, in turn, may adversely affect development in two ways: by altering cellular differentiation of the developing fetus and by reducing blood flow to and from the placenta. Decreased blood flow would be expected to result in a lowered supply of amino acids and oxygen to the fetus. Both decreased oxygen and decreased nutrient supply would have a negative effect on growth and development.

Technological advances now available to the researcher will permit direct quantification of placental and umbilical blood flow and oxygen consumption. If, for example, blood flow is found to be reduced in alcohol-consuming pregnant animals, scientists can begin to address the biochemical reason for the decreased flow, such as an imbalance of certain prostaglandins.

Scientists also are using animal models to examine the effects of alcohol on chemical growth factors, which play a critical role in the regulation of cell growth and survival, and on other biological mechanisms that may be involved in programmed death of brain cells (West et al. 1990). Fetal development is a complex process that itself is not yet fully understood. Cell signaling systems that appear to control various aspects of fetal development, such as certain neurotransmitters (e.g., *N*-methyl-d-aspartate [NMDA] and serotonin), retinoic acid, and cyclic AMP, may have a significant role in FAS, as may the expression of developmental genes. The cell signaling systems and the expression of developmental genes both appear to be affected by alcohol (Pullarkat and Azar 1991; Hogan 1991).

## Conclusion

Many more potential mechanisms of action remain. This area of investigation into the basis for the teratogenic actions of alcohol is still in its infancy stage. The future looks bright for animal research to make major advances not only in the identification of potential mechanism(s) but also in the evaluation of various types of interventions. As for other medical conditions, the development of appropriate treatment strategies for FAS can only be realized and successfully implemented when the mechanisms underlying the myriad defects related to fetal alcohol exposure are uncovered.

## Figures and Tables

**Table 1:** Behavioral Effects Following Prenatal Alcohol Exposure in Humans and Animals

Humans	Animals
Hyperactivity	Increased activity and exploration
Attention deficits, distractibility	Attention deficits
Lack of inhibition	Lack of inhibition
Mental retardation, learning difficulties	Impaired learning
Impaired ability to adjust to new stimuli or situations (impaired habituation)	Impaired habituation
Repetition of a mental activity with an inability to switch to another activity (perseveration)	Perseveration
Feeding difficulties	Feeding difficulties
Gait abnormalities	Gait abnormalities
Poor fine and gross motor skills	Poor coordination
Developmental delay (motor, social, and language development)	Developmental delay (motor reflexes, behavior, puberty)
Hearing abnormalities	Hearing abnormalities
Poor state regulation (tremors, jitters, aberrant sleep patterns)	Poor state regulation (temperature dysregulation, aberrant sleep patterns)

SOURCE: Adapted from Driscoll et al. 1990.

## Footnotes

1 The effects observed in females are more variable and less clear.

2 This is a hypothesized mechanism that is supported by several research findings (see NIAAA 1990 see NIAAA 1993). It may not be simply due to a static increase or decrease in prostaglandin levels but rather to a change in the *balance* of these compounds.
